# Exploring spatio-temporal changes in coastal recreational fisheries and potential links to extreme weather events

**DOI:** 10.1371/journal.pone.0305106

**Published:** 2024-06-07

**Authors:** Faith A. Ochwada-Doyle, Nathan Miles, Julian M. Hughes, Jeffrey J. Murphy, Michael B. Lowry, Laurie West, Matthew D. Taylor

**Affiliations:** 1 New South Wales Department of Primary Industries, Sydney Institute of Marine Science, Mosman, NSW, Australia; 2 Kewagama Research, Doonan, Queensland, Australia; 3 New South Wales Department of Primary Industries, Narrandera, NSW, Australia; 4 New South Wales Department of Primary Industries, Wollongong, New South Wales, Australia; 5 New South Wales Department of Primary Industries, Port Stephens, New South Wales, Australia; 6 University of New South Wales, Kensington, New South Wales, Australia; University of Ferrara, ITALY

## Abstract

Extreme weather events across coastal environments are expected to increase in frequency under predicted climate change scenarios. These events can impact coastal recreational fisheries and their supporting ecosystems by influencing the productivity of fish stocks or altering behaviours and decision-making among fishers. Using off-site telephone/diary survey data on estuarine and oceanic recreational fishing activity in eastern Australia, we analyse interannual and geographic variability in bream (*Acanthopagrus* spp) and snapper (*Chrysophrys auratus*) catch, total effort and total catch per unit effort (CPUE) through a period (2013/2014, 2017/2018 and 2019/2020) that encompassed severe drought, bushfires and flooding. Interacting spatial and temporal differences were detected for bream and may reflect spatial variation in the intensity and extent of some of the extreme weather events. The catch of snapper did not change temporally, providing little evidence that this species’ catch may be influenced by the extreme weather events. Independent bioregional and temporal effects on effort were detected, while CPUE only showed significant bioregional differences. Although adverse conditions created by the extreme weather events may have dissuaded fisher participation and impacted effort, we propose that the observed temporal patterns in effort reflect the early influence of socio-economic changes brought on by the COVID-19 pandemic on coastal recreational fishing, over and above the impacts of extreme weather events. This study demonstrates how interrelated ecological, social and economic factors can shape coastal recreational fisheries and facilitates development of management strategies to address future threats to the sector.

## Introduction

The frequency and intensity of extreme weather events such as hurricanes, heat waves, drought, floods and wildfires and is predicted to increase under projections of climate change [1, 2]. Although direct attribution of extreme weather events to anthropogenic climate change is a topic of ongoing scientific debate [3, 4], the trend of increased extreme events has recently become apparent in many parts of the world [5–7]. These events can have significant and measurable impacts on coastal recreational fisheries, which shape the ecology and play vital socio-economic roles in the marine resource landscape of many high-income and transitional nations [8, 9]. Like many other factors, extreme weather events can influence the productivity of fish stocks or alter behaviours and decision-making among fishers and thereby impact recreational fishery metrics such as catch and catch per unit effort (CPUE) [10, 11].

Coastal recreational fisheries can be directly or indirectly affected by extreme weather events. Direct effects include preclusion of fishery access through bad weather or damage to fishing infrastructure such as shorelines, marinas, boats and fishing gear; as well as loss of fish biomass due to displacement or mortality. For instance, an extreme cold event in southern Florida during 2010 resulted in widespread fish-kills for the common snook, *Centropomus undecimalis* [12]. This species supports a substantial coastal recreational fishery that experienced subsequent reductions in catch per unit effort within western estuaries [12]. Indirect effects of extreme weather events on coastal recreational fisheries can include the loss of coastal habitats such as marshes, oyster reefs and mangroves which support the productivity of exploited fish populations. Duke, Kovacs [13] described how extreme high temperatures and low precipitation contributed to extensive dieback of mangrove tidal vegetation in Australia’s Gulf of Carpentaria and inferred a reduction in harvest for recreational fisheries targeting marine finfish (e.g., king salmon (*Polydactylus macrochir*)) that depend on mangrove habitats.

Monitoring recreational fisheries and integrating dynamic management actions in response to extreme weather events can provide social and economic benefits to resource users, support ecosystem benefits and promote fisheries sustainability [14, 15]. Recreational fishing surveys are useful tools for monitoring temporal variation in fishery indices, particularly as the balance of effort in fisheries shifts among sectors and recreational catches of many species exceed that of other sectors. However, the large, diffuse and heterogenous nature of many recreational fisheries can make it difficult to collect reliable and representative on-site data in a cost-effective way [16]. Offsite telephone/diary surveys based on randomized sampling overcome many of these challenges by enabling broad yet efficient fishery coverage due to larger sample sizes and/or higher response rates [17–19]. Such surveys are consequently used to routinely monitor and assess expansive recreational fisheries in nations such as the USA, Germany and Australia [20–23].

In the Australian state of New South Wales (NSW), off-site telephone/diary surveys have been implemented over the last decade to track spatio-temporal patterns of recreational fishing activity [24, 25]. NSW is home to the nation’s greatest number of recreational fishers, with participation rates of approximately 11.9% [21, 26]. The coastal saltwater fishery consists of estuarine and oceanic components across 2,000 km of coastline and is the largest in the state [26, 27]. The recreational fisheries of NSW have been monitored using a standardised annual survey design applied only in 2013/14; 2017/18 and 2019/20. The last two surveys coincided with periods during which three extreme weather events occurred in NSW, potentially impacting coastal aquatic environments [28, 29]: (i) the most severe drought recorded in the European history of Australia [30]; (ii) the “Black Summer” bushfires which burnt through more land than any fires in the past 25 years [5, 6]; and, (iii) heavy rainfall and subsequent flooding that led to some of the highest river levels since 1992 [7]. The synchronicity of these extreme weather events with the two later surveys provided a unique opportunity to examine changes in annual recreational fishery metrics in relation to the extreme weather events.

This study therefore reports on temporal patterns in coastal recreational fishing activity through 2013/14, 2017/18 and 2019/20 to understand shifts in the fishery that coincided with the extreme weather events. Focusing on the line-based recreational fishery (also known as angling) of NSW, which represents the dominant (97% of participation) form of fishing [24, 31], this study specifically compares estuarine and oceanic fishing effort, CPUE and the species-specific total catch of *Acanthopagrus* spp (species complex of *A*. *butcheri* [black bream] and *A*. *australis* [yellowfin bream], hereafter referred to as bream) and *Chrysophrys auratus* (hereafter referred to as snapper) across the study period. These species are consistently among the five most commonly caught species in both the estuarine and oceanic waters of NSW [24, 31] and are also of significant commercial importance within the state, contributing ~ $2M per annum in production value [32–34]. Furthermore, both species are predominantly captured using line-based fishing in NSW [24, 31]. Since the extent of the extreme weather events in question was not spatially homogenous and recreational activity in NSW has been shown to vary spatially [25, 35], this study also examines whether coarse spatial interactions occurred with temporal patterns of recreational fishing. This research explores how interrelated ecological, social and economic drivers can potentially shape coastal marine recreational fisheries in a rapidly changing world.

## Materials and methods

This study, involving human participants, was approved by the New South Wales Department of Primary Industries Human Ethics process (INT20/76587).

### Study area

**Fig 1:**
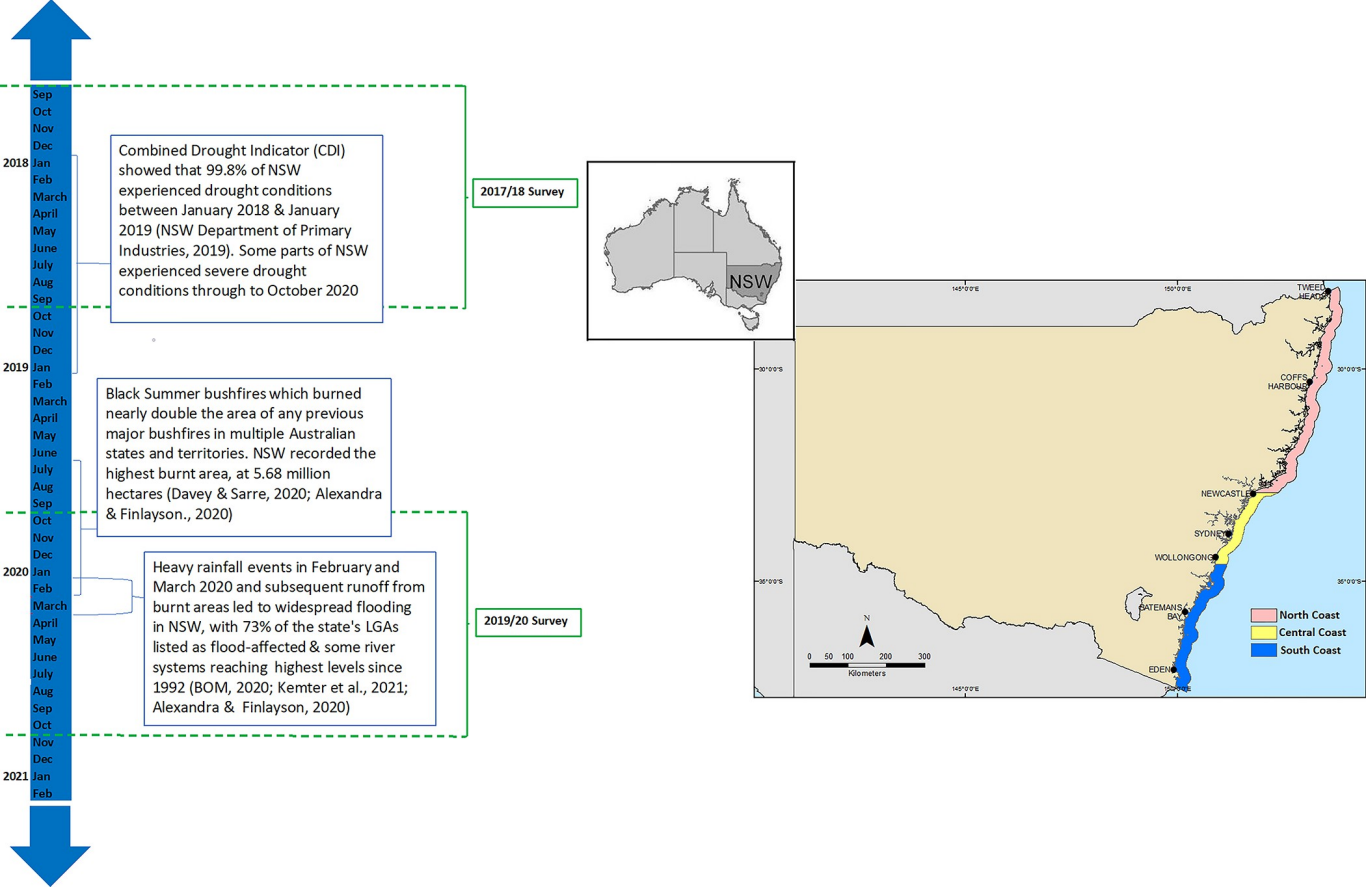
Map of New South Wales (NSW), Australia indicating the relative sizes of the North Coast, Central Coast and South Coast regions. The state boundary shown in the map was generated by Geoscience Australia as part of the Australian Marine Spatial Information System (https://amsis-geoscience-au.hub.arcgis.com/) and the Bioregion boundaries are generated from the Integrated Marine and Coastal Regionalisation of Australia v4.0 (https://fed.dcceew.gov.au/datasets/erin::integrated-marine-and-coastal-regionalisation-of-australia-imcra-v4-0-provincial-bioregions/about). The source maps are released under the Creative Commons Attribution (4.0 and 3.0 respectively) International Licence. Included is a timeline showing the sequence of extreme weather events that took place within NSW during the 2017/18 and 2019/20 recreational fishing surveys. Note that a preceding 2013/14 survey is not included in the timeline.

This study considered estuarine and oceanic recreational fishing data collected across the NSW coastline (also known as the NSW Marine Estate; NSW Marine Estate Management Authority [36]). This coastline is divided into 3 geographic management bioregions by the state’s Marine Estate Management Authority: the North Coast (which extends from the Queensland border (28.16° S; 153.55° E) down to Stockton Beach (32.81° S; 151.96°E)); the Central Coast (which extends from Stockton Beach to Shellharbour (34.58° S; 150.87°E)); and the South Coast (which extends from Shellharbour to the Victorian boarder (37.51° S; 149.98° E)) [36, 37] (Fig 1). For this study, estuarine waters were defined as saltwater estuaries, bays and inlets while oceanic waters were those that spanned from the coastline to the 200 nm exclusive economic zone (Fig 1). In this context, coastal waters encompass both estuarine and oceanic environments. The 3 geographic management areas were affected by severe drought, bushfires and flooding to varying degrees during the 2013/14, 2017/18 and 2019/20 periods. Table 1 presents a qualitative comparison of the relative intensity of each of these extreme weather events in each geographic area in each year. Although the COVID-19 pandemic was not an extreme weather event of initial interest in this study, it was an unprecedented socio-economic shock that took place during the 2019/20 and, as discussed later, had the potential to influence the fishery metrics of interest as was reported in other parts of the world [38, 39]. Its relative intensity is therefore also depicted in Table 1.

**Table 1:** Qualitative comparison of the relative intensity of each severe drought, bushfires and floods that occurred during the 2013/14, 2017/18 and 2019/20 periods in each of the North (N), Central (C) and South (S) Coasts of NSW, Australia. Also shown is the relative intensity of the COVID-19 Pandemic. The intensity of each event is categorised as either negligible (green); low (yellow); moderate (orange); or, high (red).

Year	Bioregion	Severe Drought	Severe Bushfires	Heavy Rainfall & Flooding	COVID-19 Pandemic
2013/14	**North**				
	**Central**				
	**South**				
2017/18	**North**				
	**Central**				
	**South**				
2019/20	**North**				
	**Central**				
	**South**				
References	** **	[40–42, 75]	[33, 42–44]	[42, 45, 46]	[47, 48]

### Data collection

Annual data on the recreational fishery was collected for fishing activity that occurred during the 2013/14, 2017/18 and 2019/20 periods. For each period, data was collected using an off-site telephone-diary survey developed to provide cost-effective state-wide fishery information over a large spatial scale [21, 26, 49]. The approach involved a two-phase design, with an initial *Screening Phase* and the second an intensive *Diary Phase*. The *Screening Phase* occurred from March to May 2013, September to October 2017 and August to October 2019. It was a structured telephone interview of a randomly selected sample of households from the NSW Recreational Fishing Licence (RFL) database of 1–3 year licence holders [31]. During the screening phase, profiling information was collected (including age, gender and fishing avidity) and respondents answered questions regarding their intention to fish in the ensuing 12 months to determine their eligibility for the *Diary Phase*. A longitudinal panel survey made up the study’s *Diary Phase* and monitored the fishing activity of all residents (aged ≥ 5 years) within recruited households between June 2013 to May 2014, between October 2017 to September 2018 and between November 2019 and October 2020 [24, 31]. Fishing information collected during the *Diary Phase* included date, location, start and finishing times, fishing methods, fishing platform (boat- or shore-based), waterbody type (freshwater, estuarine or oceanic), target species and numbers of kept and released animals by species/species group [31]. This information was recorded by trained interviewers during regular telephone calls to diarists (which were made on at least a monthly basis to reduce recall bias) [31]. The *Screening Phase* and *Diary Phase* are described in greater detail in Ochwada-Doyle, Miles [50] and Murphy, Ochwada-Doyle [31].

### Data analysis

The statistical programming software *R* [51] was used for all analyses and graphing described hereafter. To enable estimation of total and mean population catch, effort, and CPUE, analysis of each year’s raw survey data was based on a stratified random survey design. The RFL holders (n) represented the primary sampling unit within a randomly selected household, and the fishing activity of all resident fishers within the household was surveyed (representing a form of single stage cluster sampling). Adjustment and calibration were integrated to expand information from each sampled household to population estimates. A response propensity model was fitted to partially-responding (assumed to be representative of non-responders) and fully-responding RFL holders who provided information on the fishing activity of household residents in the last 12 months. This model (which predicted a fisher’s unique combination of characteristics that are expected to affect the relative likelihood of obtaining a complete survey response from that fisher [52, 53]) was then used to adjust sample data for response bias [26, 53]. Calibration (weighting adjustment to make the estimated and known population totals consistent with each other) also implemented using benchmark data (known population total of RFL holders) containing the total number of licenced holders in each of the state’s ten residential strata [26, 53, 54]. In order to account for unexpected “drop-ins” into the fishery, an additional adjustment was applied based on a “non-intending call-back” survey. This accounted for the avidity index (which was measured for household residents aged ≥5 yrs. as a function of estimated fishing frequency in the previous 12 months [53]) reported for “drop-ins” and residential stratum [26, 53]. The *Survey* [54] package was used to expand and calibrate the raw data and provide total population estimates of species-specific catch and total combined effort following the approach outlined in Lyle *et al*. [53] (see Lyle, Wotherspoon and Stark [53] and Lumley [54] for a full description and equations). Mean population estimates of species-specific catch and total effort were also generated from the expanded raw data on species-specific catch and total combined effort. The raw data on total catch across all species and total combined effort were used to calculate total CPUE as a function of numbers caught/fisher day for each fisher. They were then expanded and used to estimate mean CPUE across RFL households.

For catch, effort and CPUE data from each survey period, separate analyses were conducted for data from oceanic and estuarine environments. Using the glmer.nb() function in the *lme4* package [55], generalized linear mixed-effects models (GLMMs) assuming a negative binomial distribution [56, 57] were used to examine whether the mean of expanded estimates of species-specific line-based catch (across retained and released catch) for each of bream and snapper (in terms of numbers) changed among the three survey years (2013/14, 2017/18 and 2019/20) and bioregions (North Coast, Central Coast and South Coast). The same analyses were used to examine whether mean expanded line-based effort (in terms of fisher days) changed among the three survey years and bioregions. It’s important to note that robust measures of effort could only be estimated across all species captured within a given fishing method due to anglers commonly targeting more than one species during a single fishing event or not specifying a target species in the first place. Similarly, mean expanded CPUE could only be estimated and analysed across all species within a given fishing method. There have been recent attempts to estimate species-specific CPUE from this data which have focused on fishing events where a fisher has identified the species of interest as a target species [58, 59]. Although this approach can be useful for generating broad performance indicators for a species’ fishery, it was not applied in the current study because it relies on the assumption that fishers report their targeting behaviour accurately after their fishing trip (i.e. the reporting is likely to be biased by fishers posteriorly identifying a species as a target species simply because they happened to catch that species in large numbers) and leads to omission of data from trips where there were no specific targets (27% of fishing trips averaged across survey years). In each of the estuarine and oceanic data sets, the line-based CPUE data (in terms of numbers caught across all species per day) had many zeros (>50% of records). The CPUE data were consequently modelled using zero-inflated Poisson GLMMs executed using the mixed_model() function in the *GLMMadaptive* package [57, 60, 61]. The use of GLMMs enabled us to account for any non-independence among primary sampling units through inclusion of random-effects terms for individual persons and households [62, 63].

The full GLMMs for catch and effort considered all independent parameters and interaction terms and took the general form of:

$$\log\left(\mu_{i}\right)=\beta_{0}+\beta_{1}x_{1,ij}+\beta_{2}x_{2,ij}+\beta_{3}x_{1,ij}x_{2,ij}+a_{1,i}{+a}_{2,j}+\varepsilon_{ij}$$ (1)

where μ_i_ equals the expected value E(*Y*_*i*_) and *Y*_*i*_ represents the observed catch or effort of a sampled angler after it has been expanded to represent the catch of all anglers in the known population represented by that sampled angler; β_0_ is the vertical intercept; β_1_ and β_2_ are the partial regression coefficients of the regressors for the categorical independent parameters year (*x*_1,*ij*_) and bioregion (*x*_2,*ij*_) respectively; β_3_ is the partial regression coefficient for their two-way interaction; *a*_*1*,*i*_ and *a*_*2*,*j*_ respectively represent the random variables person and household; and, ε_ij_ represents the error term [56, 64, 65].

The full zero-inflated GLMMs for CPUE considered all independent parameters and interactions and took on the general form of:

$$\log\left(\mu_{i}\right)=\beta_{0}+\beta_{1}x_{1,ij}+\beta_{2}x_{2,ij}+\beta_{3}x_{1,ij}x_{2,ij}+a_{1,i}{+a}_{2,j}+\varepsilon_{ij}$$

$$logit(\pi )=\gamma_{0}+{\gamma_{1}x}_{1,ij}+{\gamma_{2}x}_{2,ij}+\gamma_{3}x_{1,ij}x_{2,ij}+v_{1,i}{+v}_{2,j}$$ (2)

In Eq 2, μ_i_(1-π) equals the expected value E(*Y*_*i*_) and *Y*_*i*_ represents the observed non-zero CPUE value of a sampled angler after it has been expanded to represent the CPUE of all anglers in the known population represented by that angler. β_0–3_, *x*_1,*ij*_, *x*_2,*ij*_, *a*_*1*,*i*_, *a*_*2*,*j*_ and ε_ij_ in the first part of Eq 2 (the count component) denote the same attributes as described for Eq 1. The second part of Eq 2 (the zero-inflated component) represents the probability of excess zeros in the data. Here π is the probability of observing a zero; *logit()* is the inverse logit function that maps the probabilities to the log-odds scale; γ_0_ is the vertical intercept; γ_1_ and γ_2_ are the partial regression coefficients of the regressors for the independent parameters year (*x*_1,*ij*_) and bioregion (*x*_2,*ij*_) respectively; γ_3_ is the partial regression coefficient for their two-way interaction;; *v*_*1*,*i*_ and *v*_*2*,*j*_ respectively represent the random effects of person and household on the probability of excess zeros [57, 65–67].

Eq 1 and the first part of Eq 2 were initially fitted assuming both a Poisson and a negative binomial distribution family. Akaike Information Criteria (AICs) values were then compared among these models to select the model that had the most appropriate distribution family, whereby the model with the lowest AIC value was deemed most appropriate [64]. Based on AIC values, the parameters in the original full model from the most suitable distribution family were iteratively reduced towards parsimony through consecutive exclusion of a parameter [62, 68, 69]. If exclusion of a particular parameter led to a higher AIC value, this meant that the model’s parsimony was not improved by that parameter’s exclusion and the parameter was therefore retained in the model [64, 70]. In doing so, the analyses retained the subset of predictor parameters that were most important in explaining variation in catch, effort or CPUE [64]. Partial tests have been shown to be an insufficient measure of the appropriateness of alternatives to a model [see 68, 71, 72], so were only used to assess the influence of an independent parameter or interaction term once the final model had been chosen. For each final parsimonious GLMM, a partial Wald test (α = 0.01) was used to examine the null hypothesis that β_i_ = 0 [55, 64, 73] for a retained parameter or the interaction term. To enable us to determine the influence of each predictor variable on both the count and zero-inflated components of the final parsimonious zero-inflated GLMMs, partial ɀ-tests (α = 0.01) were used to examine the null hypotheses that β_i_ = 0 or γ_i_ = 0 [55, 64, 73] for a retained parameter or the interaction term. Where no significant interactions were present and the influence of region was shown to be independently significant, Tukey’s post-hoc tests (α = 0.01) were then used to compare the different levels of region in a pair-wise manner with the interaction term from the original model being used as the error term [64]. The same procedure was used where no significant interactions were present, and the influence of year was shown to be independently significant. Wherever a parsimonious GLMMs showed that year and bioregion had a significant interaction, a separate secondary series of GLMMs were applied to examine the influence of year at each level of region separately using Wald tests or ɀ-tests (α = 0.01) [55, 64, 74]. This examination was followed by Tukey’s post-hoc tests (α = 0.01) whenever year was found to be significant at a particular level of region.

## Results

The sample sizes (n = number of RFL holders) used in the survey expansion procedure to estimate the catch, effort or CPUE for a particular year within each bioregion are shown in Table 2. These values also represent the sample size for each year within a region in the GLMMs.

**Table 2:** The sample sizes (n = number of long-term Recreational Fishing Licence holders) used in the survey expansion procedure to generate population estimates of catch, effort or catch per unit effort (CPUE) for a particular year within the North Coast (N), Central Coast (C) and South Coast (S) estuarine and oceanic waters of NSW (Australia) for the line-based recreational fishery.

	2013/14			2017/18			2019/20		
Estuarine Waters	**N**	**C**	**S**	**N**	**C**	**S**	**N**	**C**	**S**
Bream catch	142	102	95	158	107	97	119	93	64
Snapper catch	14	62	37	9	48	55	11	50	32
Total effort	577	559	589	566	544	568	461	424	451
Total CPUE	577	559	589	566	544	568	461	424	451
Oceanic Waters	**N**	**C**	**S**	**N**	**C**	**S**	**N**	**C**	**S**
Bream catch	57	26	33	66	23	23	57	13	25
Snapper catch	51	21	47	50	23	44	43	17	31
Total effort	483	486	466	467	447	448	364	358	325
Total CPUE	483	486	466	467	447	448	364	358	325

### Bream catch

**Fig 2:**
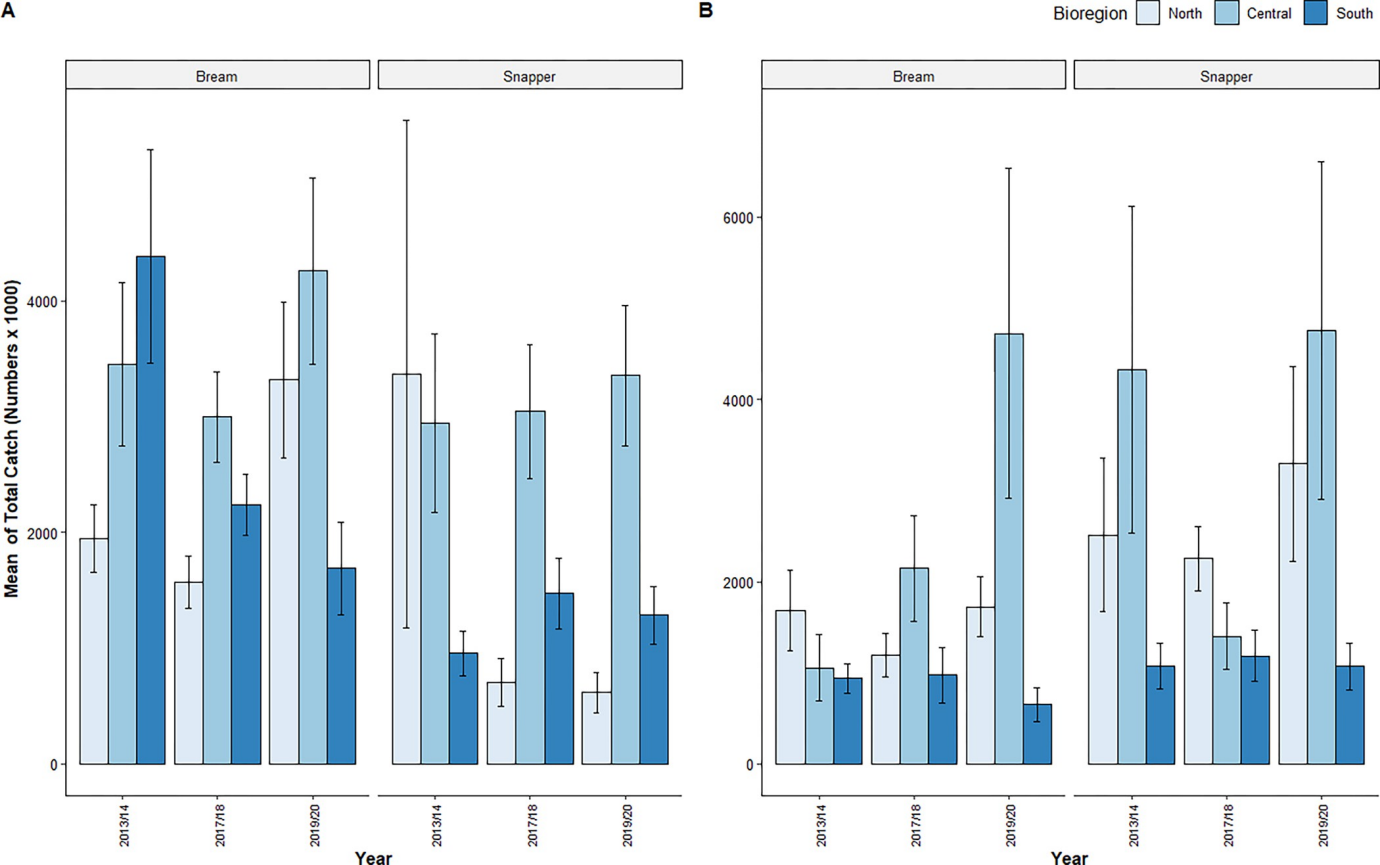
Mean of estimated total (retained and released) number (x 1000) of bream and snapper caught in (a) estuarine and (b) oceanic waters through line-based recreational fishing activity in NSW in 2013/14, 2017/18 and 2019/20. Error bars represent 1 standard error of the mean estimated catch.

For bream caught within estuarine waters, the most parsimonious GLMM retained both year and bioregion as well as their interaction term, which was significant (Table 3). The secondary GLMMs examining the influence of year at each level of bioregion separately showed that year influenced catch in the North Coast and South Coast (North Coast: DF = 2, P(>|Chi^2^|) < 0.001, n = 419; Central Coast: DF = 2, P(>|Chi^2^|) = 0.08, n = 302; South Coast: DF = 2, P(>|Chi^2^|) < 0.001, n = 256). For estuarine waters in the North Coast, Tukey’s post-hoc test revealed that total catch of bream was highest in 2019/20 followed by 2013/14 followed by 2017/18 (Table 4; Fig 2A). In the South Coast, the total catch of bream showed a sequential decline between 2013/14 and 2019/20 (Table 4; Fig 2A).

**Table 3:** Results of the generalized linear mixed effects models used to examine the influence of year (3 levels: 2013/14, 2017/18 and 2019/20) and region (3 levels: North Coast, Central Coast and South Coast) on the estimated total number of bream and snapper caught (retained and released) through line-based recreational fishing activity in the estuarine and oceanic waters of NSW during 2013/14, 2017/18 and 2019/20. The models, which assumed a negative binomial distribution, applied the Wald test (α = 0.01) to examine the null hypothesis that a β_i_ = 0, where β_i_s were the regression coefficients for region, year or their interaction. The total sample sizes (n = number of long-term Recreational Fishing Licence holders) of each model are also shown.

**Bream catch**							
**Estuary (n = 977)**				**Ocean (n = 323)**			
	**Chi** ^**2**^	**DF**	**P(>|Chi** ^**2**^ **|)**		**Chi** ^**2**^	**DF**	**P(>|Chi** ^**2**^ **|)**
Region	28.11	2	<0.001***	Region	8.06	2	0.01*
Year	9.88	2	0.007**	Year	2.92	2	0.23
Region:Year	19.77	4	<0.001***	Region:Year	12.21	4	0.01*
**Snapper catch**							
**Estuary (n = 318)**				**Ocean (n = 327)**			
	**Chi** ^**2**^	**DF**	**P(>|Chi** ^**2**^ **|)**		**Chi** ^**2**^	**DF**	**P(>|Chi** ^**2**^ **|)**
Region	18.95	2	<0.001***	Region	18.65	2	<0.001***
Year	4.12	2	0.13	Year	1.11	2	0.57

**Table 4:** The results of Tukey’s post-hoc tests (α = 0.01) comparing different levels of year (3 levels: 2013/14, 2017/18 and 2019/20) or region (3 levels: North Coast, Central Coast and South Coast) in terms of the total catch of bream, total catch of snapper, total effort and total catch per unit effort (CPUE) as estimated for line-based recreational fishing in the estuarine and oceanic waters of NSW during 2013/14, 2017/18 and 2019/20.

**Bream catch**							
Estuary–North Coast				Ocean–Central Coast			
	**Estimate**	**SE**	**P(>|ɀ|)**		**Estimate**	**SE**	**P(>|ɀ|)**
2017/18–2013/14 = 0	-0.73	0.16	<0.001***	2017/18–2013/14 = 0	0.54	0.09	<0.001***
2019/20–2013/14 = 0	1.14	0.16	<0.001***	2019/20–2013/14 = 0	1.31	0.12	<0.001 ***
2019/20–2017/18 = 0	1.87	0.04	< 2E-16 <0.001***	2019/20–2017/18 = 0	0.77	0.15	8.74E-07 <0.001***
Estuary–South Coast				Ocean–South Coast			
	**Estimate**	**SE**	**P(>|ɀ|)**		**Estimate**	**SE**	**P(>|ɀ|)**
2017/18–2013/14 = 0	-0.29	0.19	0.38	2017/18–2013/14 = 0	-0.09	0.12	1
2019/20–2013/14 = 0	-0.87	0.19	<0.001***	2019/20–2013/14 = 0	-0.25	0.09	0.01*
2019/20–2017/18 = 0	-0.59	0.02	<0.001***	2019/20–2017/18 = 0	-0.34	0.08	<0.001***
**Snapper Catch**							
Estuary				Ocean			
	**Estimate**	**SE**	**P(>|ɀ|)**		**Estimate**	**SE**	**P(>|ɀ|)**
North—Central = 0	-0.63	0.25	0.01*	North—Central = 0	-0.04	0.19	1
South—Central = 0	-0.62	0.15	<0.001***	South—Central = 0	-0.69	0.21	<0.001***
South—North = 0	0.01	0.26	1	South—North = 0	-0.65	0.16	1.77E-04***
**Total Effort**							
Estuary				Ocean			
	**Estimate**	**SE**	**P(>|ɀ|)**		**Estimate**	**SE**	**P(>|ɀ|)**
North—Central = 0	-0.09	0.02	<0.001***	North—Central = 0	-0.09	0.03	0.009**
South—Central = 0	-0.06	0.02	0.01*	South—Central = 0	0.07	0.03	0.06
South—North = 0	-0.03	0.02	0.23	South—North = 0	-0.02	0.03	1
Estuary				Ocean			
	**Estimate**	**SE**	**P(>|ɀ|)**		**Estimate**	**SE**	**P(>|ɀ|)**
2017/18–2013/14 = 0	-0.07	0.07	0.93	2017/18–2013/14 = 0	-0.07	0.06	0.76
2019/20–2013/14 = 0	0.15	0.07	0.07	2019/20–2013/14 = 0	0.13	0.07	0.18
2019/20–2017/18 = 0	0.22	0.04	<0.001***	2019/20–2017/18 = 0	0.20	0.05	<0.001***
**Total CPUE**							
Estuary				Ocean			
	**Estimate**	**SE**	**P(>|ɀ|)**		**Estimate**	**SE**	**P(>|ɀ|)**
North—Central = 0	-1.92	0.10	<0.001***	North—Central = 0	0.44	0.16	0.01*
South—Central = 0	0.64	0.10	0.24	South—Central = 0	1.50	0.26	<0.001***
South—North = 0	2.56	0.11	<0.001***	South—North = 0	1.06	0.23	<0.001***

For oceanic waters, the most parsimonious total catch model for bream also retained both independent parameters and their interaction term, which was significant (Table 3). The secondary GLMMs showed that year influenced oceanic catch in the Central Coast and South Coast (North Coast: DF = 2, P(>|Chi^2^|) = 0.05, n = 180; Central Coast: DF = 2, P(>|Chi^2^|) < 0.001, n = 62; South Coast: DF = 2, P(>|Chi^2^|) < 0.001, n = 81). For the Central Coast, total catch of bream was highest in 2019/20 followed by 2017/18 followed by 2013/14 (Table 4; Fig 2B). In the South Coast, the total catch of bream was similarly high in 2013/14 and 2017/18 followed by 2019/20 (Table 4; Fig 2B).

### Snapper catch

The most parsimonious GLMM for snapper caught in estuarine waters retained both year and bioregion but excluded their interaction term. Bioregion was the only significant parameter (Table 3) and Tukey’s post-hoc tests showed that the total catch of snapper was significantly greater in the Central Coast compared to both the South Coast and North Coast (Table 4; Fig 2A).

The most parsimonious model on oceanic snapper catch also retained both independent parameters and excluded their interaction term. The only significant parameter was bioregion (Table 3) and Tukey’s post-hoc tests showed that the total oceanic catch of snapper was significantly lower in the South Coast compared to the North Coast and Central Coast (Table 4; Fig 2B).

### Total effort

**Fig 3:**
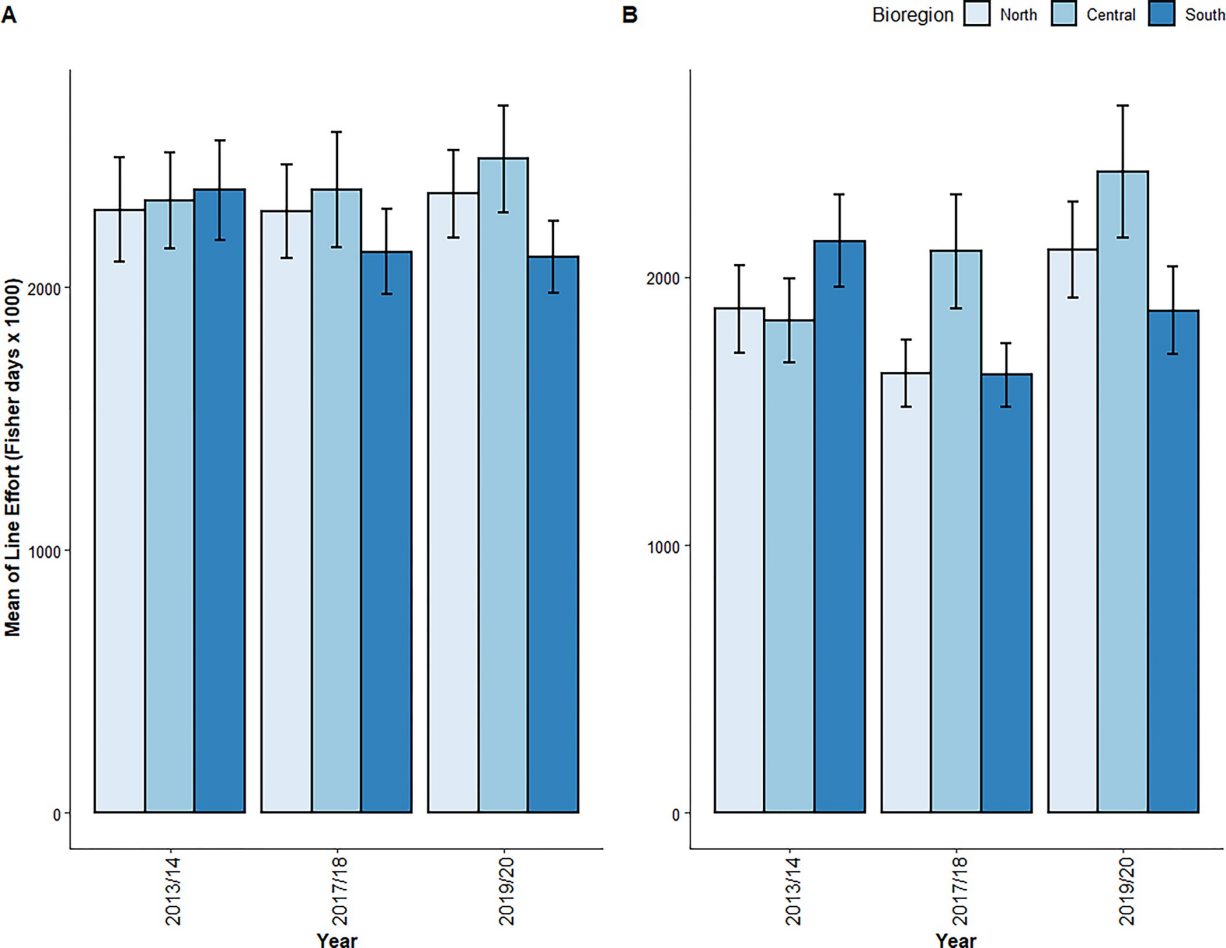
Mean of estimated total effort (fisher days x 1000) expended in (a) estuarine and (b) oceanic waters through line-based recreational fishing activity in NSW in 2013/14, 2017/18 and 2019/20. Error bars represent 1 standard error of the mean estimated effort.

The most parsimonious GLMM for total line-based effort in estuarine waters retained both year and bioregion but excluded their interaction term. Both bioregion and year influenced effort significantly (Table 5). Tukey’s post-hoc tests showed that effort, pooled across years, was significantly greater in the Central Coast compared to both the South Coast and North Coast (Table 4, Fig 3A). Temporally, the only significant difference in effort was between 2019/20 and 2017/18 with the former year having the greatest effort (Table 4, Fig 3A).

**Table 5:** Results of the generalized linear mixed effects models used to examine the influence of year (3 levels: 2013/14, 2017/18 and 2019/20) and region (3 levels: North Coast, Central Coast and South Coast) on the estimated total fishing effort (fisher days) expended through line-based recreational fishing activity in the estuarine and oceanic waters of NSW during 2013/14, 2017/18 and 2019/20. The models, which assumed a negative binomial distribution, applied the Wald test (α = 0.01) to examine the null hypothesis that a β_i_ = 0, where β_i_s were the regression coefficients for region or year. The total sample sizes (n = number of long-term Recreational Fishing Licence holders) of each model are also shown.

Total effort							
Estuary (n = 4,739)				Ocean (n = 3,844)			
	**Chi** ^**2**^	**DF**	**P(>|Chi** ^**2**^ **|)**		**Chi** ^**2**^	**DF**	**P(>|Chi** ^**2**^ **|)**
Region	24.09	2	<0.001***	Region	9.68	2	0.008***
Year	26.08	2	<0.001***	Year	13.40	2	0.001***

For oceanic data on total effort, the most parsimonious model also retained both independent parameters and excluded their interaction term. Both year and bioregion were significant (Table 5). Tukey’s post-hoc tests showed that, spatially, the Central Coast had greater effort than the North Coast and, temporally, 2019/20 had greater effort than 2017/18 (Table 4; Fig 3B).

### Total CPUE

**Fig 4:**
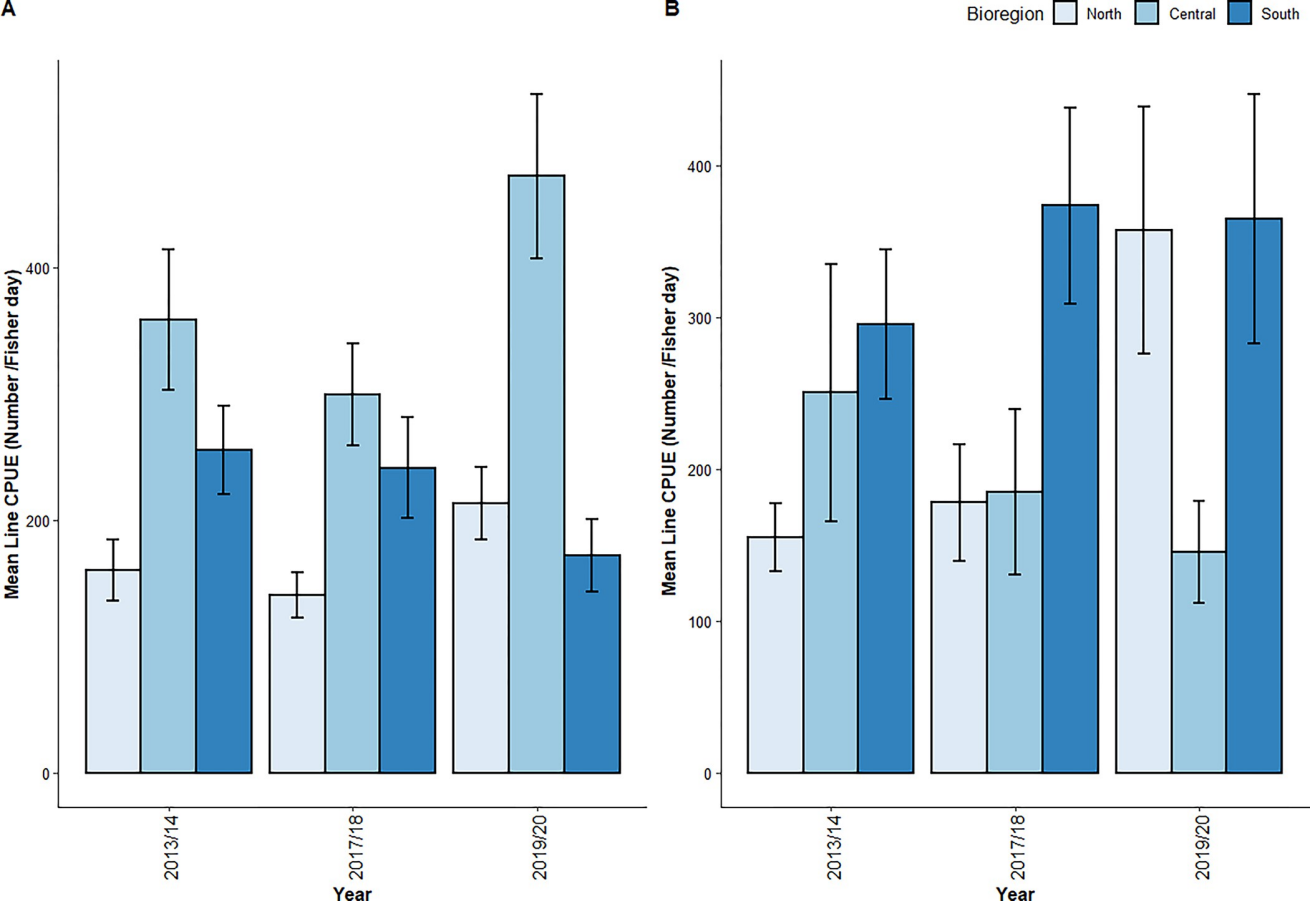
Mean of estimated total catch per unit effort (CPUE; number/fisher days x 1000) for (a) estuarine and (b) oceanic line-based recreational fishing in NSW in 2013/14, 2017/18 and 2019/20. Error bars represent 1 standard error of the mean estimated CPUE.

The most parsimonious zero-inflated GLMM for total line-based CPUE in estuarine waters retained both year and bioregion but excluded their interaction term. Only bioregion had a significant influence on CPUE for both the count and zero-inflated components of the model (Table 6). Tukey’s post-hoc tests showed that CPUE, pooled across years, was significantly greater in the Central Coast and the South Coast compared to the North Coast (Table 4; Fig 4A).

**Table 6:** Results of the zero-inflated Poisson generalized linear mixed effects models used to examine the influence of year (3 levels: 2013/14, 2017/18 and 2019/20) and region (3 levels: North Coast, Central Coast and South Coast) on the catch per unit effort (CPUE; numbers/fisher days) estimated for line-based recreational fishing activity in the estuarine and oceanic waters of NSW during 2013/14, 2017/18 and 2019/20. To enable examination of the influence of each predictor variable on both the count and zero-inflated components of each model, partial ɀ-tests (α = 0.01) were used to examine the null hypotheses that β_i_ = 0 or γ_i_ = 0 for a retained parameter or interaction term (where β_i_ and γ_i_ respectively represent the partial regression coefficients for a variable’s regressor in the count and zero-inflated components of the model). The total sample sizes (n = number of long-term Recreational Fishing Licence holders) of each model are also shown.

Total CPUE									
Estuary (n = 4,739)					Ocean (n = 3,844)				
Count component									
	**β**_**i**_	**SE**	**ɀ-value**	**P(>|ɀ|)**		**β**_**i**_	**SE**	**ɀ-value**	**P(>|ɀ|)**
Intercept	0.42	0.11	3.70	<0.001***	Intercept	-1.07	0.18	-5.83	<0.001***
Region_North_	-1.92	0.10	-19.57	<0.001***	Region_North_	0.44	0.16	2.70	0.007***
Region_South_	0.64	0.13	4.83	<0.001***	Region_South_	1.50	0.26	5.78	<0.001***
Year_2017/18_	-0.07	0.12	-0.58	0.56					
Year _2019/20_	0.07	0.12	0.57	0.57					
Zero-inflated component									
	**γ**_**i**_	**SE**	**ɀ-value**	**P(>|ɀ|)**		**γ**_**i**_	**SE**	**ɀ-value**	**P(>|ɀ|)**
Intercept	-0.47	0.21	-2.26	0.02	Intercept	-0.10	0.23	-0.44	0.66
Region_North_	-6.76	1.16	-5.84	<0.001***	Region_North_	-0.98	0.24	-4.16	<0.001***
Region_South_	1.05	0.22	4.86	< 1.00E-04***	Region_South_	0.09	0.28	0.30	0.76
Year_2017/18_	-0.09	0.21	-0.42	0.68					
Year _2019/20_	0.10	0.22	0.45	0.65					

The most parsimonious zero-inflated GLMM for total line-based CPUE in oceanic waters excluded year and the interaction term and retained bioregion, which had a significant influence on CPUE for both the count and zero-inflated components of the model (Table 6). Tukey’s post-hoc tests showed that CPUE, pooled across years, was significantly greater in the South Coast followed by the North Coast followed by the Central Coast (Table 4; Fig 4B).

## Discussion

Extreme whether events resulting from increased sea and atmospheric temperatures as well as changes in precipitation can impact coastal recreational fisheries and their supporting ecosystems by influencing the productivity of fish stocks or altering behaviours and decision-making among fishers [10, 11]. This research monitored interannual variability in bream and snapper catch, total effort and total CPUE across an entire jurisdiction’s estuarine and oceanic waters through a period that encompassed severe drought, bushfires and flooding. Each of these extreme weather events impacted different geographic regions and waterbodies variably, with our results suggesting some interactive effects and spatial heterogeneity in terms of fishery metrics.

Discussed below are a selection of the spatio-temporal differences in fishery metrics observed in relation to potential explanatory socio-ecological factors (see Table 7 for summary). While some of the changes in fishery metrics may be linked to the extreme weather events; others are more plausibly explained by considering other factors such as the changes brought about by the COVID-19 pandemic. We stress that there are an array of inter-related mechanisms that could contribute to the fishery patterns observed and therefore pose our explanations as potential links only. To enable less equivocal inferences about the exact causes of the observed spatio-temporal variation, we recommend future experimental work that includes unaffected control sites (for examining localised extreme whether events such floods) that could enable application of a before-after-control-impact (BACI) analytical design [75]. A longer time-series (>20 years) of survey data including explicit questions on how often extreme weather events affected a person’s choice to fish, angling behaviour and perceived success may also provide robust insights into causes of variation.

**Table 7:** Summary of the main inter-annual differences in fishery metrics observed in this study and a brief description of the socio-ecological factors that may explain each observation.

Point	Inter-annual difference	Potential explanatory socio-ecological factor
(i)	Temporal differences in estuarine bream catch in the South Coast (2013/14 = 2017/18>2019/20) vs North Coast (2019/20>2013/14>2017/18)	Erosion caused by more intense bushfires in the South Coast compared to the North Coast during 2019/20 may have impacted benthic habitats of bream contributing to greater mortality for this species in this region.
(ii)	Temporal differences in oceanic bream catch in the Central Coast (2019/20>2017/18>2013/14) vs South Coast (2013/14 = 2017/18>2019/20)	The 2018–2019 drought conditions decreased streamflow more severely in the Central Coast and may have triggered downstream migration of bream into oceanic habitats. This may have increased abundance and catchability in the Central Coast resulting in the greater oceanic recreational landings observed in this region.
(iii)	Temporal difference in recreational effort in both estuarine and oceanic waters (2019/20>2017/18)	The increase in effort observed for 2019/20 may reflect the influence of changes caused by the COVID-19 pandemic on coastal recreational fishing. The pandemic resulted in a substantial portion of the NSW coastal metropolitan populations working from home and other portions experiencing job losses or reduced work hours, all of which may have increased participation in recreational fishing.

The survey showed that for bream caught in estuaries in the North Coast, catches were highest in 2019/20 followed by 2013/14 and lowest in 2017/18 (Table 7; point i). However, the number of bream caught in estuaries in the South Coast was significantly lower during 2019/20 (Table 7; point i). One explanation for these spatio-temporal differences in the estuarine catch of bream may be linked to disparities in the intensity and extent of the bushfires. The bushfires of the austral spring and summer 2019/20 affected NSW’s coastal regions variably, with the South Coast showing the greatest quantity of canopy affected by fire from the area just south of Shellharbour through to Bega (~157km north of Victorian boarder) [35]. After the fires, the south-east corner of NSW experienced some of the largest percentage increases in erosion due to reduced soil stability—a common consequence of bushfires [35]. Bushfires can rapidly impact estuaries through such erosion, especially when coupled with post-fire rainfall, and can thus influence estuary hydrology, turbidity and nutrient inputs via sedimentation. Benthic habitats including seagrass beds and shellfish reefs [28].which are important are important habitats for bream [76, 77], can be affected by this sedimentation [28]. The intensity of the bushfires in the south-east may have therefore reduced the habitat available to bream and contributed to their mortality in this region to a greater extent, possibly resulting in the lower catches during 2019/20. Silva, Doyle [78] identified *A*. *butcheri* (black bream) during assessments of aquatic biota mortalities within South Coast waterways following the 2019/20 fires. It is also possible that the change in water quality simply led to species such as bream avoiding fire-impacted estuaries in the South Coast, thus leading to lower catches.

For ocean caught bream, total catch in the Central Coast was lowest 2013/14 with moderate to high recreational catches estimated for 2017/18 and 2019/20, respectively (Table 7; point ii). In the South Coast however, the oceanic catch of bream was similarly high in 2013/14 and 2017/18 and lowest in 2019/20 (Table 7; point ii). The drought conditions recorded between 2018 to early 2019 resulted in reduced water in the soil, rivers and groundwater across much of Australia but had contrasting effects on streamflow from south to north in eastern Australia [79]. These conditions are one of many possible factors that may have contributed to the differences observed in oceanic bream catch. In NSW, the drought was shown to decrease coastal streamflow more severely in the Central and North Coast compared to the South Coast and may partly explain the opposing patterns observed for oceanic bream catch in the Central and South Coast [79]. This north-ward decline in streamflow is likely to have increased estuarine salinity in the Central and North Coast and could have triggered downstream migration into oceanic habitats for marine-estuarine opportunist species like bream [29, 80]. Accordingly, Gillson, Suthers and Scandol [29] showed that NSW commercial landings of *A*. *australis* (yellowfin bream) were greater in oceanic habitats during drought due to an increase in their abundance and thus catchability following estuarine emigration. A similar phenomenon may have occurred with oceanic recreational landings in the Central Coast following the drought period of 2018 to early 2019.

Although the interaction between region and year was not deemed important in explaining variation in fishing effort in either estuarine or oceanic waters, independent bioregional and temporal effects were found. When temporal effects were examined without accounting for bioregional effects, 2019/20 consistently showed increased levels of effort when compared to 2017/18 (Table 7; point iii). Based on trends reported for the state’s freshwater recreational fishery and in other parts of Australia [39, 50], it was expected that adverse fishing conditions caused by the bushfires and flooding, in particular, might cause a decline in coastal effort by 2019/20. In contrast, the high effort observed for 2019/20 here may reflect the early influence of socio-economic changes caused by the COVID-19 pandemic on coastal recreational fishing, over and above the impacts of the extreme weather events. As governments tried to control the spread of COVID-19, measures were taken in early 2020 (and enforced from March in Australia) that restricted human movement, led to temporary cessation of non-essential activities, such as tourism, and slowed many local economies [81, 82]. In NSW, these changes resulted in high proportions of coastal metropolitan populations working from home and other portions experiencing job losses or reduced work hours. With less time committed to work or commuting, fishers in NSW may have increased their engagement with the relatively safe (i.e. socially isolated) and low-cost outdoor activity of recreational fishing, as was reported in other parts of the world where fishing activity exceeded pre-pandemic levels [38, 83, 84].

For estuarine and oceanic snapper catch as well as overall CPUE, inter-annual variation was not detected, providing little evidence that these metrics were influenced by the extreme weather events for this species. Adult snapper are generally found in offshore oceanic waters [85], where environmental perturbations caused by events such as bushfires, droughts and floods are likely to be of less consequence. Although extreme weather events can impact the estuarine and near-shore nursery environments inhabited by juvenile snapper [85], past research suggests that juvenile snapper are well-adapted to the shifts in salinity, turbidity and oxygen availability [86–88] brought about by extreme events. Snapper may have therefore had a greater resilience and likelihood of recruiting into recreational fisheries in both estuarine and oceanic environments despite the extreme weather events. CPUE could only be confidently estimated across all species caught using line-based methods (~92 individual species/taxa). The inclusion of such a large number of species with divergent patterns of habitat-use, biology and differing responses to factors like extreme weather events may have introduced substantial noise into the catch component of CPUE and therefore made it challenging to detect interannual variation. Overcoming this limitation in the future could involve quantifying species-specific CPUE through customized surveys that do not rely on broad assumptions about targeting behaviour.

## Conclusion

This study shows interesting variation in fishery metrics in a coastal recreational fishery that may be driven by socio-ecological dynamics. It also highlights the utility of off-site surveys of recreational fishers in monitoring this variability. As climate change and related extreme weather events continue to affect the world’s coastal environments, fisheries are likely to be impacted and will require ongoing adaptive management to ensure their sustainability. Quantitative monitoring studies can contribute critical data to assist in identifying problematic trends and in making projections. This, in turn, facilitates the development of targeted management strategies to address future threats to recreational fisheries [89, 90]. Such strategies may include habitat enhancement through the deployment of purpose-built artificial reef systems in estuaries affected by sedimentation to protect bream populations in regions prone to bushfires and floods [91]. Another targeted management strategy could involve the introduction of water management plans that determine and implement environmental flow requirements specific to estuarine ecosystems and manage these flows holistically from catchment to coast [92]. This strategy might ensure adequate stream flow for fish in estuaries within drought affected regions.

## Supporting information

S1 Data (ZIP)

## References

[1] Barange M, Bahri T, Beveridge MC, Cochrane KL, Funge-Smith S, Poulain F. Impacts of climate change on fisheries and aquaculture: synthesis of currrent knowledge, adaptation and mitigation options: fao; 2018.

[2] Alimonti G, Mariani L, Prodi F, Ricci RA. A critical assessment of extreme events trends in times of global warming. The European Physical Journal Plus. 2022;137(1):112.

[3] Zanocco C, Boudet H, Nilson R, Satein H, Whitley H, Flora J. Place, proximity, and perceived harm: extreme weather events and views about climate change. Clim Change. 2018;149(3):349–65.

[4] Stott PA, Christidis N, Otto FEL, Sun Y, Vanderlinden J-P, van Oldenborgh GJ, et al. Attribution of extreme weather and climate-related events. WIREs Climate Change. 2016;7(1):23–41. doi: 10.1002/wcc.380 26877771 PMC4739554

[5] Rural Fire Service. Bushfire Bulletin. Journal of the NSW Rural Fire Service. 2020;42(1).

[6] Alexandra J, Finlayson CM. Floods after bushfires: rapid responses for reducing impacts of sediment, ash, and nutrient slugs. Australasian Journal of Water Resources. 2020;24(1):9–11.

[7] Kemter M, Fischer M, Luna LV, Schönfeldt E, Vogel J, Banerjee A, et al. Cascading hazards in the aftermath of Australia’s 2019/2020 Black Summer wildfires. Earth’s Future. 2021;9:1–7.

[8] Arlinghaus R, Cooke SJ, Sutton SG, Danylchuk AJ, Potts W, Freire KdMF, et al. Recommendations for the future of recreational fisheries to prepare the social-ecological system to cope with change. Fish Manag Ecol. 2016;23(3–4):177–86.

[9] Holder PE, Jeanson AL, Lennox RJ, Brownscombe JW, Arlinghaus R, Danylchuk AJ, et al. Preparing for a changing future in recreational fisheries: 100 research questions for global consideration emerging from a horizon scan. Rev Fish Biol Fish. 2020;30(1):137–51.

[10] Brander KM. Global fish production and climate change. Proceedings of the National Academy of Sciences. 2007;104(50):19709–14. doi: 10.1073/pnas.0702059104 18077405 PMC2148362

[11] Townhill BL, Radford Z, Pecl G, van Putten I, Pinnegar JK, Hyder K. Marine recreational fishing and the implications of climate change. Fish and Fisheries. 2019;20(5):977–92.

[12] Stevens PW, Blewett DA, Boucek RE, Rehage JS, Winner BL, Young JM, et al. Resilience of a tropical sport fish population to a severe cold event varies across five estuaries in southern Florida. Ecosphere. 2016;7(8):e01400.

[13] Duke NC, Kovacs JM, Griffiths AD, Preece L, Hill DJE, van Oosterzee P, et al. Large-scale dieback of mangroves in Australia’s Gulf of Carpentaria: a severe ecosystem response, coincidental with an unusually extreme weather event. Mar Freshw Res. 2017;68(10):1816–29.

[14] Hunt LM, Fenichel EP, Fulton DC, Mendelsohn R, Smith JW, Tunney TD, et al. Identifying Alternate Pathways for Climate Change to Impact Inland Recreational Fishers. Fisheries. 2016;41(7):362–72.

[15] Sainsbury NC, Genner MJ, Saville GR, Pinnegar JK, O’Neill CK, Simpson SD, et al. Changing storminess and global capture fisheries. Nature Climate Change. 2018;8(8):655–9.

[16] Pollock KH, Jones CM, Brown TL. Angler survey methods and their applications in fisheries management. Bethesda: American Fisheries Society; 1994. 371 p.

[17] Beckmann C, Tracey S., Murphy J., Moore A., Cleary B. and Steer M., editor. Assessing new technologies and techniques that could improve the cost-effectiveness and robustness of recreational fishing surveys.2019.

[18] Bellanger M, Levrel H. A cost-effectiveness analysis of alternative survey methods used for the monitoring of marine recreational fishing in France. Ocean & Coastal Management. 2017;138:19–28.

[19] Georgeson L, Moore A, Ward P, Stenekes N, Kancans R, Mazur K, et al. A framework for regular national recreational fishing surveys. Canberra: Australian Bureau of Agriculture and Resource Economics and Sciences; 2015. Contract No.: ABARES project 43534

[20] Lynch TP, Smallwood CB, Ochwada-Doyle F, Williams J, Ryan KL, Devine C, et al. A cross continental scale comparison of Australian offshore recreational fisheries research and its application to marine park and fisheries management. ICES J Mar Sci. 2020;77(3):1190–205.

[21] Henry GW, Lyle JM. The national recreational and indigenous fishing survey. Canberra, Australia: Australian Government Department of Agriculture, Fisheries & Forestry; 2003. Report No.: 158.

[22] Dorow M, Arlinghaus R. A telephone-diary-mail approach to survey recreational fisheries on large geographic scales, with a note on annual landings estimates by anglers in Northern Germany. Am Fish Soc Symp. 2011;75:319–44.

[23] Brick JM, Andrews W, Mathiowetz N. A comparison of recreational fishing effort survey designs. Maryland: National Marine Fisheries Service; 2012.

[24] Murphy JJ, Ochwada-Doyle FA, West LD, Stark KE, Hughes JM, Taylor MD. Survey of recreational fishing in NSW, 2019/20—Key Results. Nelson Bay: Department of Primary Industries (NSW Government); 2022. Contract No.: NSW DPI—Fisheries Final Report Series No. 161.

[25] Ochwada-Doyle F, Stark K, Hughes J, Murphy J, Lowry M, West L. Temporal and regional variation in catch across an extensive coastal recreational fishery: Exploring the utility of survey methods to guide and assess spatio-temporal management initiatives. PloS one. 2021;16(7):e0254388. doi: 10.1371/journal.pone.0254388 34288950 PMC8294510

[26] West LD, Stark KE, Murphy JJ, Lyle JM, Ochwada-Doyle FA. Survey of recreational fishing in New South Wales and the ACT, 2013/14. Port Stephens, Australia: New South Wales Department of Primary Industries; 2015. Report No.: 149 Contract No.: NSW DPI—Fisheries Final Report Series No. 149.

[27] Short AD, Woodroffe CD. The Coast of Australia. Cambridge: Cambridge University Press; 2009.

[28] Barros TL, Bracewell SA, Mayer-Pinto M, Dafforn KA, Simpson SL, Farrell M, et al. Wildfires cause rapid changes to estuarine benthic habitat. Environ Pollut. 2022;308:119571. doi: 10.1016/j.envpol.2022.119571 35661807

[29] Gillson J, Suthers I, Scandol J. Effects of flood and drought events on multi-species, multi-method estuarine and coastal fisheries in eastern Australia. Fish Manag Ecol. 2012;19(1):54–68.

[30] Bureau of Meteorology. Drought conditions in Australia and impact on water resources in the Murray-Darling Basin. Commonwealth of Australia; 2020 13/08/2020.

[31] Murphy JJ, Ochwada-Doyle FA, West LD, Stark KE, Hughes JM. Survey of recreational fishing in NSW, 2017/18. Nelson Bay: Department of Primary Industries (NSW Government); 2020. Contract No.: NSW DPI—Fisheries Final Report Series No. 158.

[32] Fowler AJ, Jackson G, Stewart J, Hamer P, Roelofs A. Snapper, *Chrysophyys auratus*. Canberra: Fisheries Research and Development Corporation; 2018.

[33] McGilvray J, Conron S, Broadhurst MK. Yellowfin bream, Acanthopagrus australis. *Canberra*: *Fisheries* Research and Development Corporation; 2018. Contract No.: Status of Australian fish stocks reports 2018.

[34] Mosby D. Australian fisheries and aquaculture statistics 2017. Fisheries Research and Development Corporation project. 2018;134:2–3.

[35] DPIE. NSW Fire and the Environment 2019–20 Summary; Biodiversity and landscape data and analyses to understand the effects of the fire events. Department of Planning, Industry and Environment, NSW; 2020.

[36] NSW Marine Estate Management Authority. NSW Marine Estate Management Strategy (2018–2028). Sydney, NSW; 2018.

[37] Jordan A, Fairfull S, Creese B. Managing threats to the marine estate in New South Wales (Australia) to maximise community wellbeing. J Coast Res. 2016(75):642–6.

[38] Howarth A, Jeanson AL, Abrams AEI, Beaudoin C, Mistry I, Berberi A, et al. COVID-19 restrictions and recreational fisheries in Ontario, Canada: Preliminary insights from an online angler survey. Fish Res. 2021;240:105961. doi: 10.1016/j.fishres.2021.105961 36540896 PMC9754797

[39] Ryan KL, Desfosses CJ, Denham AM, Taylor SM, Jackson G. Initial insights on the impact of COVID-19 on boat-based recreational fishing in Western Australia. Marine Policy. 2021;132:104646. doi: 10.1016/j.marpol.2021.104646 34602712 PMC8462792

[40] Industries NDoP. NSW State Seasonal Update—December 2019 2024 [Available from: https://www.dpi.nsw.gov.au/climate-landing/ssu/december-2019.

[41] Hatfield-Dodds S, Hughes N, Cameron A, Miller M, Jackson T. Analysis of 2018 drought; 26 October 2018. Canberra; 2018.

[42] Meteorology Bo. New South Wales in 2014: the warmest year on record 2024 [Available from: http://www.bom.gov.au/climate/current/annual/nsw/archive/2014.summary.shtml.

[43] Heritage NEa. Recovering from the 2019–20 fires 2024 [Available from: https://www.environment.nsw.gov.au/topics/fire/park-recovery-and-rehabilitation/recovering-from-2019-20-fires.

[44] Authority NEP. Fire 2024 [Available from: https://www.soe.epa.nsw.gov.au/all-themes/land/fire.

[45] Department of Planning IaE, NSW. State of the beaches 2019–2020. Parramatta: Environment, Energy and Science; 2020.

[46] Observatory NE. Extreme Rain Douses Fires, Causes Floods in Australia 2024 [Available from: https://earthobservatory.nasa.gov/images/146284/extreme-rain-douses-fires-causes-floods-in-australia.

[47] Wang S, Liu Y, Hu T. Examining the Change of Human Mobility Adherent to Social Restriction Policies and Its Effect on COVID-19 Cases in Australia. International Journal of Environmental Research and Public Health. 2020;17(21):7930. doi: 10.3390/ijerph17217930 33137958 PMC7662641

[48] Huveneers C, Jaine FRA, Barnett A, Butcher PA, Clarke TM, Currey-Randall LM, et al. The power of national acoustic tracking networks to assess the impacts of human activity on marine organisms during the COVID-19 pandemic. Biol Conserv. 2021;256:1–13. doi: 10.1016/j.biocon.2021.108995 34580542 PMC8457752

[49] Lyle JM, Coleman AP, West L, Campbell D, Henry GW. New large-scale survey methods for evaluating sport fisheries. Recreational fisheries: ecological, economic and social evaluation. 2002;15:207–26.

[50] Ochwada-Doyle F, Miles N, Murphy J, Stark KE, Lowry M, West LD, et al. Interannual variation in a freshwater recreational fishery under the influence of drought, bushfires, floods and a global pandemic. Mar Freshw Res. 2023;In Press.

[51] R Core Team. R: A language and environment for statistical computing. COmputing RFfS, editor. Vienna, Austria 2017.

[52] Lavrakas PJ, Jackson M, McPhee C. The use of response propensity modeling (RPM) for allocating differential survey recruitment strategies: purpose, rationale and implementation. Survey Practice. 2018;11(2).

[53] Lyle JM, Wotherspoon S, Stark KE. Developing an analytical module for large-scale recreational fishery data based on phone-diary survey methodology. Fisheries Research and Development Corporation; 2010. Contract No.: FRDC Project No.2007/064.

[54] Lumley T. Complex surveys: a guide to analysis using R. New Jersey: John Wiley & Sons; 2010.

[55] Bates D, Maechler M, Walker S, Christensen R, Singmann H, Dai B, et al. Package ’lme4’. 2020.

[56] Coelho R, Infante P, Santos MN. Comparing GLM, GLMM, and GEE modeling approaches for catch rates of bycatch species: A case study of blue shark fisheries in the South Atlantic. Fish Oceanogr. 2020;29(2):169–84.

[57] Maunder MN, Punt AE. Standardizing catch and effort data: a review of recent approaches. Fish Res. 2004;70(2–3):141–59.

[58] Fowler AM, Dowling NA, Lyle JM, Alós J, Anderson L, Cooke S, et al. Toward sustainable harvest strategies that include recreational fishing. Fish and Fisheries. 2023;In Review.

[59] Fowler AM, Ochwada-Doyle FA, Dowling NA, Folpp H, Hughes JM, Lowry MB, et al. Integrating recreational fishing into harvest strategies: linking data with objectives. ICES J Mar Sci. 2022;79:285–307.

[60] Rizopoulos D. Package ‘GLMMadaptive’. 2022.

[61] Terraube J, Van doninck J, Helle P, Cabeza M. Assessing the effectiveness of a national protected area network for carnivore conservation. Nature Communications. 2020;11(1):2957. doi: 10.1038/s41467-020-16792-7 32528022 PMC7289803

[62] Morrongiello JR, Thresher RE. A statistical framework to explore ontogenetic growth variation among individuals and populations: a marine fish example. Ecol Monogr. 2015;85(1):93–115.

[63] Zuur AF, Ieno EN, Walker NJ, Saveliev A, Smith GM. Mixed effects models and extensions in ecology with R. New York: NY: Springer; 2009.

[64] Quinn GP, Keough KJ. Experimental Design and Data Analysis for Biologists. Cambridge: Cambridge University Press; 2002.

[65] Fox J. Generalized linear models. Applied regression analysis and generalized linear models. California: Sage; 2008. p. 379–424.

[66] Hedeker D, Gibbons RD. Application of random-effects pattern-mixture models for missing data in longitudinal studies. Psychological methods. 1997;2(1):64.

[67] Lambert D. Zero-inflated Poisson regression, with an application to defects in manufacturing. Technometrics. 1992;34(1):1–14.

[68] Boyce MS, Vernier PR, Nielsen SE, Schmiegelow FK. Evaluating resource selection functions. Ecol Model. 2002;157:281–300.

[69] Field SA, Tyre AJ, Thorn KH, O’Connor PJ, Possingham HP. Improving the efficiency of wildlife monitoring by estimating detectability: a case study of foxes (*Vulpes vulpes*) on the Eyre Peninsula, South Australia. Wildl Res. 2005;32:253–8.

[70] Ochwada FA, Scandol JP, Gray CA. Predicting the age of fish using general and generalized linear models of biometric data: a case study of two estuarine finfish from New South Wales, Australia. Fish Res. 2008;90:187–97.

[71] Anderson DR, Burnham KP, Thompson WL. Null hypothesis testing: problems, prevalence, and an alternative. The journal of wildlife management. 2000:912–23.

[72] Burnham KP, Anderson DR. Practical use of the information-theoretic approach. Model selection and inference: Springer; 1998. p. 75–117.

[73] Duursma R, Powell J. Mixed-effects models. In: Duursma R, Powell J, editors. Data analysis and visualisation with R. Penrith2016. p. 3–44.

[74] Underwood AJ. Experiments in ecology: their logical design and interpretation using analysis of variance. Cambridge: Cambridge University Press; 1997. 504 p.

[75] Underwood AJ. Beyond BACI: the detection of environmental impacts on populations in the real, but variable, world. J Exp Mar Biol Ecol. 1992;161(2):145–78.

[76] Taylor MD, Becker A, Lowry MB. Investigating the Functional Role of an Artificial Reef Within an Estuarine Seascape: a Case Study of Yellowfin Bream (Acanthopagrus australis). Estuaries and Coasts. 2018;41(6):1782–92.

[77] Martinez-Baena F, Raoult V, Taylor MD, Gaston TF, McLeod I, Bishop MJ. Trophic Structure of Temperate Australian Oyster Reefs Within the Estuarine Seascape: a Stable Isotope Analysis. Estuaries and Coasts. 2023;46(3):844–59.

[78] Silva LG, Doyle KE, Duffy D, Humphries P, Horta A, Baumgartner LJ. Mortality events resulting from Australia’s catastrophic fires threaten aquatic biota. Global Change Biology. 2020;26(10):5345–50. doi: 10.1111/gcb.15282 32677160

[79] Bureau of Meteorology. Climate of the 2018–19 Financial Year 2019 [Available from: http://www.bom.gov.au/climate/updates/articles/a034.shtml.

[80] Gillson J, Scandol J, Suthers I. Estuarine gillnet fishery catch rates decline during drought in eastern Australia. Fish Res. 2009;99(1):26–37.

[81] Fernández-González R, Pérez-Pérez MI, Pérez-Vas R. Impact of the COVID-19 crisis: Analysis of the fishing and shellfishing sectors performance in Galicia (Spain). Mar Pollut Bull. 2021;169:112463. doi: 10.1016/j.marpolbul.2021.112463 34051517 PMC9751442

[82] Storen R, Corrigan N. COVID-19: a chronology of state and territory government announcements (up until 30 June 2020). Canberra: Department of Parliamentary Services, Parliament of Australia; 2020. Contract No.: Research paper series, 2020–21, 22nd October 2020.

[83] Midway SR, Lynch AJ, Peoples BK, Dance M, Caffey R. COVID-19 influences on US recreational angler behavior. PLOS ONE. 2021;16(8):e0254652. doi: 10.1371/journal.pone.0254652 34407076 PMC8372955

[84] Sbragaglia V, Brownscombe JW, Cooke SJ, Buijse AD, Arlinghaus R, Potts WM. Preparing recreational fisheries for the uncertain future: An update of progress towards answering the 100 most pressing research questions. Fish Res. 2023;263:106662.

[85] Rees MJ, Knott NA, Hing ML, Hammond M, Williams J, Neilson J, et al. Habitat and humans predict the distribution of juvenile and adult snapper (Sparidae: Chrysophrys auratus) along Australia’s most populated coastline. Estuar Coast Shelf Sci. 2021;257:107397.

[86] Fielder DS, Allan GL, Pepperall D, Pankhurst PM. The effects of changes in salinity on osmoregulation and chloride cell morphology of juvenile Australian snapper, Pagrus auratus. Aquaculture. 2007;272(1):656–66.

[87] Cumming H. Effects of turbidity on the aerobic physiology and feeding behaviour of juvenile snapper (Pagrus auratus) [Masters]: The University of Aukland; 2016.

[88] Cook DG, Iftikar FI, Baker DW, Hickey AJR, Herbert NA. Low-O2 acclimation shifts the hypoxia avoidance behaviour of snapper (Pagrus auratus) with only subtle changes in aerobic and anaerobic function. J Exp Biol. 2013;216(3):369–78. 23038727 10.1242/jeb.073023

[89] Jeanson AL, Lynch AJ, Thiem JD, Potts WM, Haapasalo T, Danylchuk AJ, et al. A bright spot analysis of inland recreational fisheries in the face of climate change: learning about adaptation from small successes. Rev Fish Biol Fish. 2021;31(2):181–200.

[90] Arlinghaus R, Abbott JK, Fenichel EP, Carpenter SR, Hunt LM, Alós J, et al. Governing the recreational dimension of global fisheries. Proceedings of the National Academy of Sciences. 2019;116(12):5209–13. 10.1073/pnas.1902796116 PMC6431172 30890655

[91] Flopp H, Schilling H, Clark G, Lowry M, Marlene B, Gregson M, et al. (2020). Artificial reefs increase fish abundance in habitat-limited estuaries. Journal of Applied Ecology. 57(9): 1752–62.

[92] Chiton D, Hamilton DP, Nagelkerken I, Cook P, Hipsey MR, Reid R, et al. (2021). Environmental flow requirements of estuaries: providing resilience to current and future climate and direct anthropogenic changes. Frontiers in Environmental Science. 9: 1–21.

